# The debate is still open; benign or malignant: a case report of a multifocal epithelioid haemangioma of fibula

**DOI:** 10.1259/bjrcr.20150269

**Published:** 2016-05-15

**Authors:** Hamid Rajebi, Shahzad Madanipour, Sahar Shiraj, Arthur Yegorov

**Affiliations:** Radiology Department, SUNY Upstate Medical University, Syracuse, NY, USA

## Abstract

In this case, we report an epithelioid haemangioma (EH) of the fibula with ill-defined multifocal lesions and a resultant pathologic fracture. Based on radiographic appearance, these lesions were initially thought to represent a malignant process, such as primary malignant bone tumour, metastases or multiple myeloma. Osseous EHs are rare. Although they can present as multifocal lesions, the majority of bony EHs are solitary and arise in the diaphysis or metaphysis of long tubular bones, with a predilection for the lower extremity. Non-specific radiological findings, debatable cytological appearance and unpredictable clinical growth patterns commonly cause misdiagnosis of malignancy. To the best of our knowledge, a case of EH with multiple growing lesions of the fibula has not yet been reported in the literature.

## Clinical presentation

A 63-year-old female with no significant prior trauma and relevant past medical history presented with a 2-month history of severe, persistent, gradually worsening, sharp and non-radiating pain over her left distal fibula. She reported that the pain interfered with sleep and did not subside with non-steroidal anti-inflammatory drugs. The pain was accompanied by no constitutional symptoms. Physical examination revealed extreme tenderness to palpation over the lateral distal fibula. There was soft tissue prominence over the mid to lower third of the fibula.

## Investigation/imaging findings

**Figure 1.**
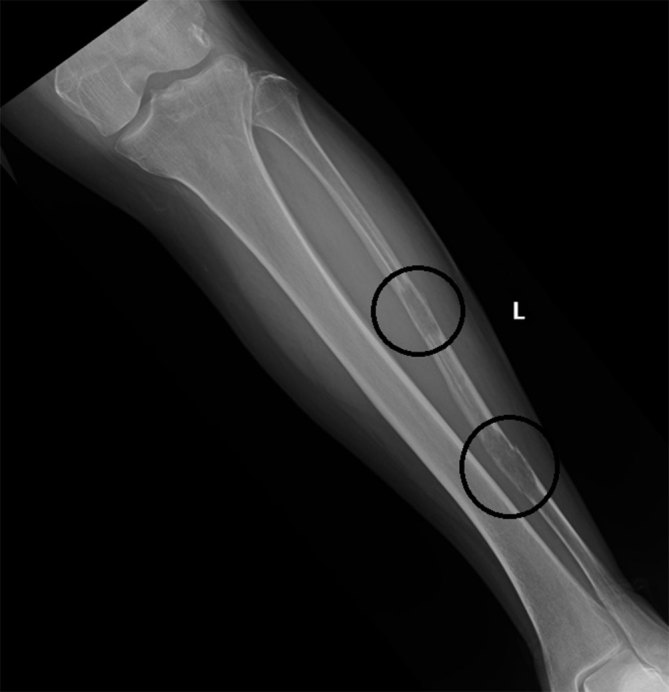
Two lytic lesions (circles) in the fibula demonstrate permeative appearance and a wide zone of transition. Differential diagnosis would include metastatic disease, multiple myeloma, lymphoma and, less likely, primary neoplasm.

**Figure 2.**
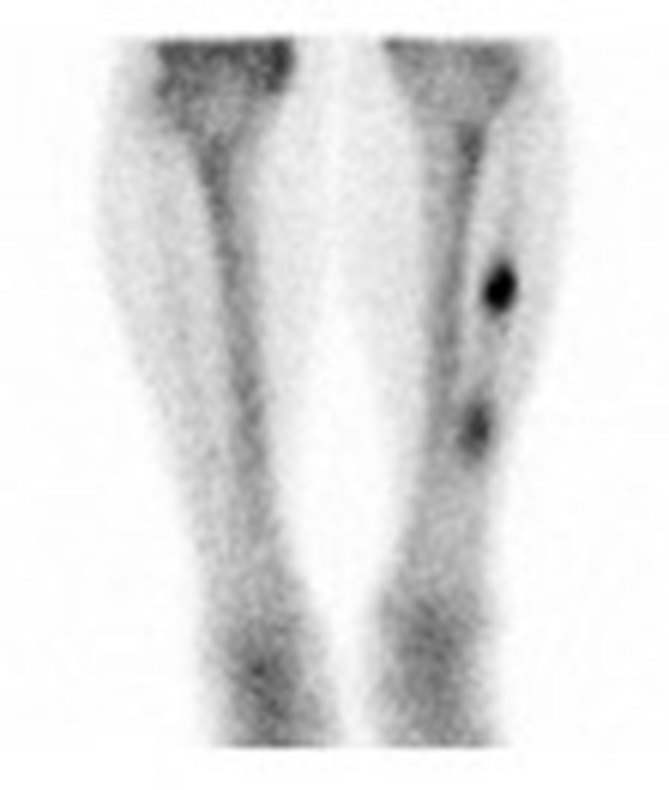
Technetium-99m methylene diphosphonate bone scintigraphy demonstrates avid radiopharmaceutical uptake within the two left fibular lesions.

Left lower leg radiographs depicted two ill-defined osteolytic destructive lesions measuring 29 and 37 mm in length in the fibular diaphysis. A pathologic fracture was present through the distal lesion (Figure 1). Bone scintigraphy was performed, which demonstrated avid radiopharmaceutical uptake within the two left fibular lesions (Figure 2). A laboratory test, including serum protein electrophoresis, alkaline phosphatase and basic metabolic profile, was ordered to evaluate for multiple myeloma. CT scans of the chest and abdomen were obtained to evaluate for primary malignancy or any evidence of metastatic disease. The results of these tests were unremarkable. Image-guided biopsy of the lesions was performed and revealed epithelioid haemangioma (EH). Immunohistochemistry analysis was positive for CD31, CD34 and factor VIII, confirming the diagnosis.

EH, also known as angiolymphoid hyperplasia with eosinophilia and histiocytoid haemangioma,^1^ is an uncommon, slow-growing vascular tumour that generally presents on the skin and subcutaneous soft tissues of the head.^2^ Osseous EHs are rare. Although they can present as multifocal lesions, the majority of bony EHs are solitary and arise in the diaphysis or metaphysis of long tubular bones, with a predilection for the lower extremity. On radiographic examination, these lesions demonstrate a well-defined osteolytic process with sclerotic margins or mixed lytic and sclerotic lesions. These lesions are often located eccentrically and may demonstrate cortical disruption or an intact cortex.^3^ In terms of histological features, intraosseous EHs have been classified as benign neoplasms in the World Health Organization taxonomy of bone tumours.^4^ However, some researchers believe that EH of bone should be considered a variant of haemangioendothelioma, a tumour with malignant potential.^5^ Non-specific radiological findings, debatable cytological appearance and unpredictable clinical growth patterns commonly cause misdiagnosis of malignancy.

## Treatment/follow-up

**Figure 3.**
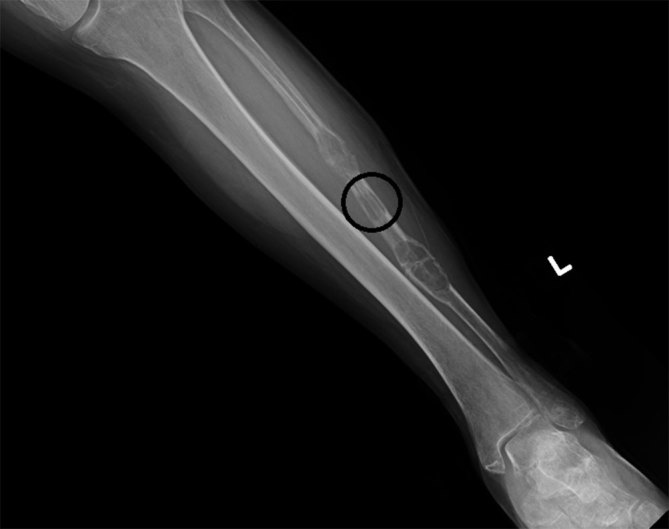
2 months later: increased size of the previous lesions with a new third lesion (circle).

**Figure 4.**
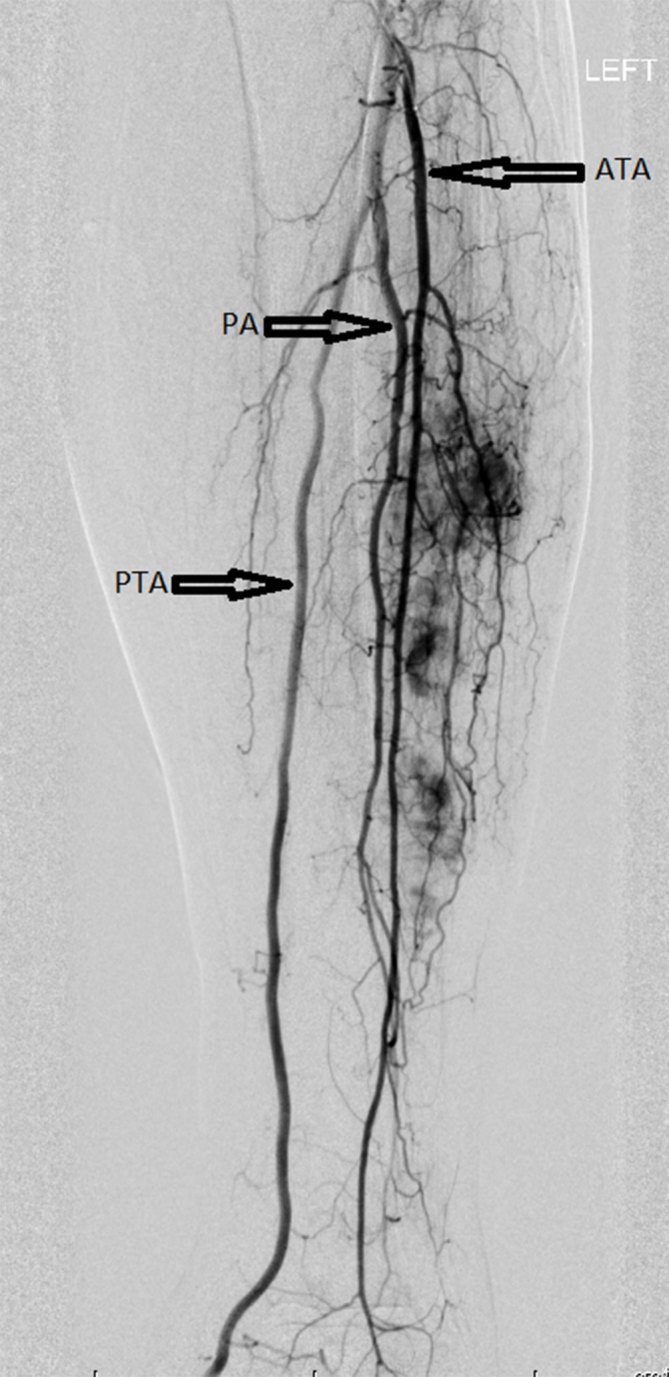
Tumour blushes of left fibular haemangiomas, with supplying branches mainly from the PA and partially from the ATA. ATA, anterior tibial artery; PA, peroneal artery; PTA, posterior tibial artery.

The patient was initially managed conservatively and placed in a walker boot. Although some pain relief was achieved, the patient’s pain persisted. At the 2-month follow-up, an additional third smaller lesion was detected along with an interval increase in size of the two previously seen lesions (Figure 3). Additional options were discussed with the patient including surgery (open resection with possible stabilization), intervention by interventional radiology (embolization of the feeding vessels of the EH), intervention by interventional radiology followed by surgery (preoperative embolization) and radiotherapy. The patient preferred to proceed with preoperative embolization followed by surgical curettage and open reduction and internal fixation. Angiograms of the left popliteal, anterior tibial and peroneal arteries showed contrast blushes in the region of the left fibular haemangiomas, with supplying branches mainly from the peroneal artery and partially from the anterior tibial artery (Figure 4). Superselection of individual feeding branches was not possible, and embolization was not performed. After unsuccessful tumour embolization, the patient underwent fibular bone lesion curettage, PRO-DENSE bone grafting (Wright Medical Technology, Inc., Memphis, TN) and open reduction and internal fixation with the left fibular diaphyseal plate. Radiographs revealed continued healing with bone graft incorporation at 1.5-, 3- and 7-month follow-ups. The patient was permitted to return to all activities without restriction 1.5 months after the surgery. She continued to improve and at the 7-month follow-up was reporting only minor pain along the lateral leg when crossing her legs. The patient ambulated independently without the need for ambulatory assistive devices.

## Discussion

Accurate diagnosis of EH requires an understanding of the clinical, radiological and pathological features of this entity. Patients can present with a wide variety of symptoms and signs, and the radiographic appearance of these lesions can mimic malignant neoplasms. The histological aspect of this group of tumours varies from lesions easily identified as being vasoformative to those that may imitate other mesenchymal neoplasms or even metastatic carcinoma.^3^

Although most osseous EHs are identified as incidental findings on radiographs, pain has been the most common presenting complaint of patients with these lesions, as in our case report. A case report of EH with secondary lymph node enlargement has been described, which demonstrates its behaviour as a malignant tumour.^6^ Females and males seem to be affected equally by EH, unlike other types of intraosseous haemangiomas that predominantly occur in females. Two cases of EH in females were reported after pregnancy, which is not the case in our patient.^7^

Intraosseous EHs lack any characteristic features and radiographs generally demonstrate a well-defined lytic lesion involving the bony metaphysis or diaphysis with associated osseous expansion and sclerosis. EHs may also show a mixed lytic and sclerotic pattern of bone destruction as described previously in the literature.^8^ EHs do not commonly cause destruction of the bony cortex but when the cortex is involved, focal cortical destruction is commonly seen with thick reactive periosteal new bone formation.^3^ Our initial radiographs demonstrated two destructive, lytic and expansile lesions with wide zones of transition associated with mild adjacent periosteal reaction. Given the patient’s clinical presentation, age and aggressive radiographic appearance, our primary differential diagnosis included metastatic disease, lymphoma, multiple myeloma and, less likely, primary bone lesions.

Histological features remain the cornerstone of the diagnosis of epithelioid vascular tumours. The differential diagnosis of EH includes epithelioid haemangioendothelioma (EHE) and epithelioid angiosarcoma. Characteristics that distinguish EH from epithelioid angiosarcoma include the absence of significant cytological atypia, brisk mitotic activity, as well as necrosis and presence of well-formed vessels.^9^ It is more difficult to differentiate between EH and EHE as a result of significant overlap at the cytological level, with epithelioid cells showing well-defined cell borders and abundantly dense eosinophilic cytoplasm. Both entities may have some level of cytological atypia. The presence of a specific myxochondroid or densely sclerotic stroma in EHE and the focal presence of mature vessels with open lumen formation in EH are the only consistent distinctive histological features between these two entities.^10^

## Learning points

1. EH is an uncommon, slow-growing vascular tumour that generally presents on the skin and the subcutaneous soft tissues of the head, with osseous EHs being rare.
2. Although osseous EHs can present as multifocal lesions, the majority of bony EHs are solitary and arise in the diaphysis or metaphysis of long tubular bones, with a predilection for the lower extremity.
3. Radiographically, EH may present as well-defined lytic lesions with sclerotic margins or mixed lytic and sclerotic lesions. These lesions are often located eccentrically and may demonstrate a disrupted or intact cortex.

## Consent

Written informed consent for the case to be published (including images, case history and data) was obtained from the patient for publication of this case report.
